# Mycosynthesized Silver Nanoparticles (AgNPs) for the Control of Foodborne Pathogenic Bacteria: *Escherichia coli* and *Staphylococcus aureus*

**DOI:** 10.3390/microorganisms14092083

**Published:** 2026-09-17

**Authors:** Mayra Eleonora Beltrán Pineda, Daniela Fernanda Valencia, José Castellanos-Rozo, Alida Marcela Gomez Rodriguez, Ingrid Rocio Fonseca-Guerra

**Affiliations:** 1 Grupo de Investigación Gestión Ambiental, Universidad de Boyacá, Tunja 150007, Colombia; danfervalencia@uniboyaca.edu.co (D.F.V.); joscastellanos@uniboyaca.edu.co (J.C.-R.); irfonseca@uniboyaca.edu.co (I.R.F.-G.); 2 Bacterióloga y Laboratorista Clínica, Universidad de Boyacá, Tunja 150007, Colombia; 3 Grupo de Investigación Núcleo, Universidad de Boyacá, Tunja 150007, Colombia; aligomez@uniboyaca.edu.co

**Keywords:** antibacterial activity, filamentous fungi, silver nanoparticles, mycosynthesis, foodborne diseases

## Abstract

This study evaluated the ability of filamentous fungi to biosynthesize silver nanoparticles (AgNPs). The nitrate reductase activity of the selected fungi, considered a key enzyme involved in AgNP biosynthesis, was assessed as a selection criterion. AgNPs were mycosynthesized using *Fusarium equiseti* AM-0197, *F. cerealis* AM-0206, *F. oxysporum* AM-0192, *Alternaria scrophulariae* AM-0187, and *Aspergillus* sp. (1-HSF-08 and 3-HSF-02) isolates. The synthesized AgNPs were characterized by transmission electron microscopy (TEM), Fourier transform infrared spectroscopy (FTIR), X-ray diffraction (XRD), and Zeta potential analysis. In all cases, spherical AgNPs were obtained, with maximum absorption peaks ranging from 400 to 420 nm. XRD analysis demonstrated a face-centered cubic crystalline structure, whereas Zeta potential measurements revealed a negative surface charge. FTIR analysis indicated the presence of organic functional groups on the nanoparticle surface acting as capping agents. The antibacterial activity of the synthesized nanomaterials was evaluated against reference strains of *Escherichia coli* and *Staphylococcus aureus*, two major foodborne pathogens, using the disk diffusion method. Inhibition zones ranged from 11.3 to 13.6 mm for *E. coli* ATCC 25922 and from 9.3 to 15.0 mm for *S. aureus* ATCC 43300. The minimum inhibitory concentration (MIC) determined by microdilution was 37.5 ppm for all AgNPs tested against *E. coli*, whereas MIC values against *S. aureus* ranged from 18.7 to 75 ppm depending on the nanoparticle evaluated. These findings demonstrate the effectiveness of mycosynthesized AgNPs as next-generation antibacterial agents for the in vitro control of foodborne bacterial pathogens.

## 1. Introduction

Foodborne diseases (FBDs) caused by the consumption of contaminated food or beverages are among the leading causes of morbidity and mortality worldwide, particularly in developing countries [1,2]. The World Health Organization (WHO) has estimated that contaminated food consumption causes approximately 600 million cases of foodborne diseases and 420,000 associated deaths annually worldwide [3].

Among the most common etiological agents of foodborne diseases are bacteria such as *Escherichia coli* and *Staphylococcus aureus*. *E. coli* is a Gram-negative bacillus that naturally inhabits the human intestine and generally does not pose a health risk. However, certain strains may acquire virulence genes through horizontal gene transfer, enabling them to cause acute gastroenteritis [4,5].

Diarrheagenic *E. coli* (DEC) is the causative agent of this disease, and strains of this species are classified into six pathotypes according to their clinical, epidemiological, and virulence characteristics: enterohemorrhagic *E. coli* (EHEC), enteropathogenic *E. coli* (EPEC), enteroaggregative *E. coli* (EAEC), enteroinvasive *E. coli* (EIEC), diffusely adherent *E. coli* (DAEC), and enterotoxigenic *E. coli* (ETEC) [6,7]. Certain enterohemorrhagic strains have been reported to cause hemolytic uremic syndrome (HUS), a severe complication affecting the kidneys and potentially leading to long-term health consequences. Therefore, rapid identification, control, and the prevention of outbreaks are essential to mitigate their impact on public health [4].

Likewise, *S. aureus* is a Gram-positive coccus that normally colonizes the skin and upper respiratory tract. Nevertheless, it is considered an opportunistic pathogen capable of causing disease in the presence of open wounds or immunosuppression [8,9]. *S. aureus* possesses a wide range of virulence factors, including exotoxins, immune evasion factors, and exoenzymes, which can cause diseases ranging from mild skin and soft tissue infections to infective endocarditis, osteomyelitis, and pneumonia [10,11].

These foodborne pathogens have been reported to harbor antibiotic resistance genes [12,13,14], thereby complicating treatment and increasing the risk of severe infections [15]. Antimicrobial resistance (AMR) is an emerging public health concern largely driven by the excessive use of antibiotics in food production, livestock farming, and agricultural practices [14]. In this context, AMR represents a major global threat, with projected annual economic losses reaching up to US$3.4 trillion by 2030, as estimated by WHO in 2025 [15].

To address AMR, nanotechnology has emerged as a promising strategy through the development of antibacterial nanomaterials that may overcome the limitations of current control protocols. Among these nanomaterials, AgNPs have attracted considerable attention owing to their remarkable efficacy against a broad spectrum of pathogens, including multidrug-resistant bacteria [16,17,18], and due to its antibacterial activity, AgNPs have been used in various applications such as food packaging, antimicrobial coatings, surface disinfection, and biomedical applications [1,16].

AgNPs can be synthesized through different physicochemical approaches, however, the development of these nanomaterials through these traditional routes presents certain limitations such as scalability, toxicity issues, and environmental impact. For this reason, the development of green synthesis or biosynthesis protocols has helped to mitigate these limitations [16]. The biological methods involve the assembly of complex molecules from simpler precursors, enabling the controlled and specific production of nanomaterials [19,20]. In biosynthetic approaches, biomolecules produced by living organisms, including plants, bacteria, and fungi, act as reducing and stabilizing agents [21,22].

Filamentous fungi have emerged as an environmentally friendly alternative for AgNP synthesis because of their ability to reduce silver compounds through the secretion of specific metabolites, including metal-reducing enzymes [20,23]. Several enzymes have been reported to participate in the synthesis process, including NADPH-dependent reductases and hydrogenases, which mediate the reduction of metallic Ag^+^ ions to elemental Ag^0^, the main constituent of AgNPs [24,25].

Several species of filamentous fungi have been successfully employed for AgNP biosynthesis, particularly species belonging to the genera *Penicillium*, *Aspergillus*, and *Fusarium* [26,27]. Fungal biosynthesis constitutes an economical and sustainable approach that allows for precise control over the size, shape, and distribution of the resulting AgNPs [27,28]. Moreover, mycosynthesized AgNPs have been reported to exhibit high biocompatibility, thereby reducing the likelihood of inducing toxicity or adverse effects in biological systems and making them suitable for a wide range of biomedical applications [21,28].

Mycosynthesized AgNPs generally exhibit potent antimicrobial properties. Therefore, the aim of the present study was to biosynthesize silver nanoparticles using filamentous fungi isolated from environmental samples and to evaluate their antibacterial activity against reference strains of *E. coli* and *S. aureus* to determine their potential as an alternative strategy for controlling foodborne bacterial pathogens.

## 2. Materials and Methods

### 2.1. Determination of Nitrate Reductase Activity in Filamentous Fungi as a Selection Criterion for AgNPs Production

Fifty filamentous fungal isolate samples from plants and rhizosphere soil and preserved in the Fungi and Microorganisms Collection of the University of Boyacá (UBCHM) were reactivated. The isolates were cultured on potato dextrose agar (PDA) medium (potato infusion, 4 g/L; glucose, 20 g/L; and agar, 15 g/L; final pH 6.0 ± 0.2). The purity and identity of each isolate at the genus or species level were confirmed according to the characteristics recorded in the identification sheet of each strain available in the collection database.

Nitrate reductase (NR) activity was subsequently evaluated by determining nitrate reduction according to a modified version of the methodology described by Winn et al. [29]. Merck^®^ nitrate broth (Darmstadt, Germany) was used as the culture medium, and the presence of nitrites was detected by adding Griess–Ilosvay reagent. The appearance of a red coloration after reagent addition was considered a positive result. To further confirm enzymatic activity, a nitrate reduction assay was performed using the colorimetric method described by Harley et al. [30]. NR activity was quantified by determining the concentration of nitrites in parts per million (ppm) present in the supernatant of inoculated liquid cultures after 7–15 days of incubation.

### 2.2. Mycosynthesis of Silver Nanoparticles (AgNPs)

The six fungal isolates exhibiting the highest NR activity, together with additional isolates showing lower enzymatic activity, were selected for AgNP biosynthesis according to the methodology described by AbdelRahim et al. [31] with minor modifications. Fungal biomass was obtained by culturing each isolate in potato dextrose broth (PDB) (potato infusion, 4 g/L; glucose, 20 g/L) under orbital shaking conditions (120 rpm) at 24 °C for 8 days. After incubation, the culture medium was filtered through sterile Whatman No. 1 filter paper, and the biomass was collected and washed three times with sterile distilled water to remove residual culture medium components and chemical traces. Subsequently, 10 g of fresh biomass was transferred to an Erlenmeyer flask containing 100 mL of Milli-Q water. The mixture was incubated under orbital shaking at 120 rpm and 24 °C for 24 h and then centrifuged at 12,000 rpm using a centrifuge Sorvall Biofuge primo R^®^ (Thermo Scientific, Osterode am Harz, Germany) for 5 min to obtain 100 mL of supernatant, designated as the mycelium-free cell filtrate (MFCF). The obtained MFCF was then mixed in equal volumes with a 3 mM AgNO_3_ solution.

The biochemical reaction between the supernatant and silver ions was carried out in the dark for 48 h at 40 °C, pH 7, and 180 rpm. The pH of the reaction was adjusted using a 1 M NaOH solution and 0.1 M HCl solution as appropriate, and using a pH meter (OAKTON Ion 510 Series ^®^, Vernon Hills, IL, USA). The bioreduction of silver ions was monitored through the color change of the reaction mixture and further confirmed by UV–Vis spectroscopy by recording spectra between 300 and 700 nm using sterile distilled water as a blank. The characteristic maximum absorption peak of silver nanoparticles at 420–430 nm was used as evidence of AgNP formation. Two controls were included: one consisting exclusively of MFCF and another containing only 3 mM AgNO_3_ dissolved in Milli-Q water without MFCF. The synthesized AgNPs were recovered by centrifugation at 5000× *g* for 5 min and subsequently characterized using different analytical techniques.

### 2.3. Characterization of the Synthesized AgNPs

The morphology and size of the synthesized AgNPs were determined by TEM. Samples were sonicated at room temperature for 10 min, and an aliquot of the aqueous AgNP suspension was deposited onto a formvar-coated copper grid and allowed to dry prior to TEM analysis [31]. The observations were performed using a Tecnai F20 transmission electron microscope (FEI Company, Hillsboro, OR, USA), operated at 100 kV. The average particle size was determined using Digital Micrograph^®^ 3 of Gatan Microscopy Suite software. The crystallinity and phase composition of the AgNPs were analyzed by XRD using a Rigaku RINT2000 vertical goniometer (Rigaku Corporation, Akishima, Tokyo, Japan), operated at 70 kV and 200 mA with Cu Kα radiation (λ = 1.5405 Å) and scanned over a 2θ range of 10–80° [20]. The average particle size of the AgNPs was calculated from the XRD pattern using Scherrer’s equation applied to the diffraction peak of the (111) plane [32]. FTIR was performed using a PerkinElmer Spectrum Two spectrometer (PerkinElmer, Waltham, MA, USA) to identify the functional groups involved in nanoparticle stabilization and surface functionalization [31]. Finally, Zeta potential measurements were performed using a Malvern Zetasizer Nano ZS instrument (Malvern Instruments Ltd., Malvern, Worcestershire, UK) to determine the colloidal stability of the AgNP suspensions [33].

### 2.4. Evaluation of the Antimicrobial Activity of Mycosynthesized AgNPs Against E. coli ATCC 25922 and S. aureus ATCC 43300

The antibacterial activity of the synthesized AgNPs was evaluated using the Kirby–Bauer disk diffusion method with minor modifications [34]. Bacterial suspensions with an optical density (OD) of 0.1 were prepared and uniformly spread onto nutrient agar plates using a sterile cotton swab. Filter paper disks (5 mm in diameter) were impregnated with a 100 ppm AgNP suspension prepared from a 300 ppm stock solution by applying the dilution equation (C_1_V_1_ = C_2_V_2_). The disks were then placed on the inoculated agar plates, and the bacterial cultures were incubated at 28 °C for 24 h. Antibacterial activity was determined by measuring the diameter (mm) of the inhibition zones surrounding each disk. Chloramphenicol (200 ppm) and trimethoprim-sulfamethoxazole (200 ppm) were used as positive controls against *E. coli* ATCC 25922 and *S. aureus* ATCC 43300 strains, respectively, whereas sterile distilled water served as the negative control. All experiments were performed in triplicate.

### 2.5. Determination of the Minimum Inhibitory Concentration (MIC) of AgNPs Against E. coli ATCC 25922 and S. aureus ATCC 43300

The minimum inhibitory concentration (MIC), defined as the lowest concentration of an antimicrobial agent capable of completely inhibiting microbial growth, was determined using the broth microdilution method according to a modified version of the Clinical and Laboratory Standards Institute guidelines [34]. Serial dilutions were prepared from a 300 ppm AgNP stock solution to obtain final concentrations of 150, 75, 37.5, 18.7, and 9.3 ppm. Briefly, 100 μL of the most concentrated antimicrobial solution was dispensed into the first well, and subsequent concentrations were added sequentially. Subsequently, 50 μL of standardized bacterial inoculum was added to each well to obtain a final bacterial concentration of approximately 5 × 10^4^ CFU/mL.

The microdilution plates were incubated at 37 °C for 24 h, and absorbance was measured at 540 nm using a Spectro Star NANO microplate reader (BMG LABTECH GmbH, Ortenberg, Germany). Inoculated nutrient broth was used as the growth control. Chloramphenicol (200 ppm) for *E. coli* and trimethoprim-sulfamethoxazole (200 ppm) for *S. aureus* were used as reference antimicrobial controls. Each concentration was tested in triplicate. MIC values were verified by assessing turbidity relative to the growth control through absorbance measurements at 540 nm. Optical density values greater than 0.5 were considered indicative of representative microbial growth.

### 2.6. Statistical Analysis

Analysis of variance (ANOVA) followed by Tukey’s multiple comparison test (α = 0.05) was performed to evaluate differences in NR activity among fungal isolates and in the antibacterial activity of the mycosynthesized AgNPs. Statistical analyses were carried out using Minitab^®^ version 19 software.

## 3. Results

### 3.1. Determination of Nitrate Reductase Activity in Filamentous Fungi as a Selection Criterion for AgNPs Production

Fifty environmental filamentous fungal isolates were successfully recovered and evaluated for their potential to synthesize AgNPs. As an initial screening approach, a qualitative assessment of nitrate reductase (NR) activity was performed. This enzyme is responsible for the reduction of Ag^+^ to elemental silver Ag^0^, the main constituent of AgNPs. Among the 50 isolates evaluated, only 18 exhibited the enzymatic activity of interest, as evidenced by the appearance of a red coloration in the culture medium after the addition of the revealing reagent. These positive isolates were subsequently subjected to quantitative analyses to confirm their enzymatic activity.

### 3.2. Quantitative Evaluation of Nitrate Reductase Activity in the Selected Fungal Isolates

**Figure 1:**
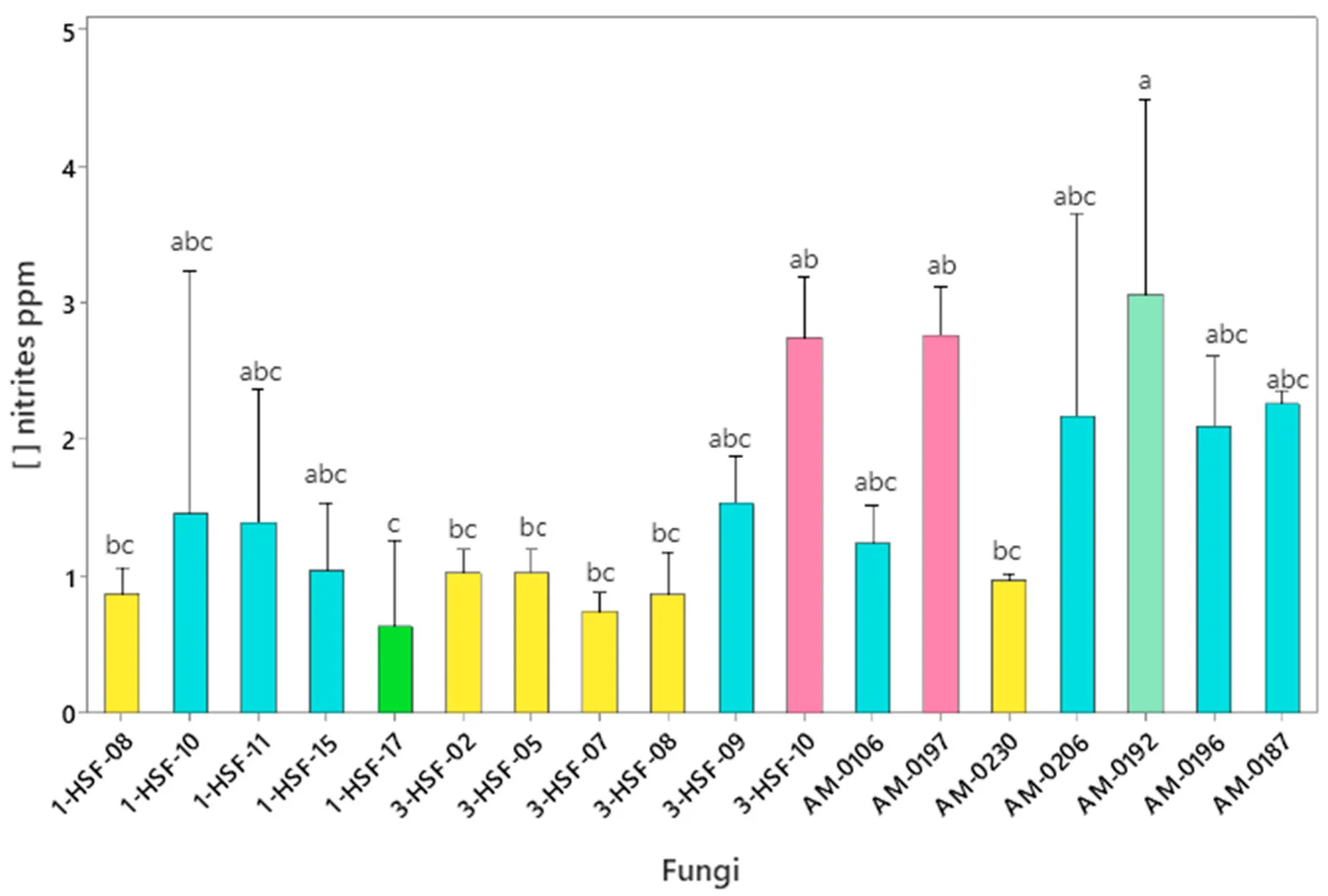
Nitrate reductase (NR) activity of promising fungal isolates from the UBCHM collection with potential for AgNP biosynthesis. Means that do not share a common letter are significantly different according to the Tukey test at 5%.

To confirm the enzymatic activity of interest in the promising isolates that tested positive during the preliminary screening, a quantitative evaluation of NR activity was performed. Among the 18 isolates evaluated, enzymatic activity, indirectly determined by the concentration of nitrite in solution, ranged from 0.635 ppm in isolate 1-HSF-17 to 3.067 ppm in isolate AM-0192 (Figure 1).

Based on these results, six fungal isolates were selected for the AgNP biosynthesis experiments. These included isolates exhibiting the highest NR activity as well as others with lower enzymatic activities in order to corroborate the importance of this criterion in a bioprospecting approach for the biosynthesis of these nanomaterials. According to the strain identification records available in the UBCHM collection database, the selected isolates were identified as *Aspergillus* sp. (1-HSF-08 and 3-HSF-02), *Fusarium cerealis* (AM-0206), *Fusarium equiseti* (AM-0197), *Fusarium oxysporum* (AM-0192), and *Alternaria scrophulariae* (AM-0187).

### 3.3. Mycosynthesis of Silver Nanoparticles (AgNPs)

**Figure 2:**
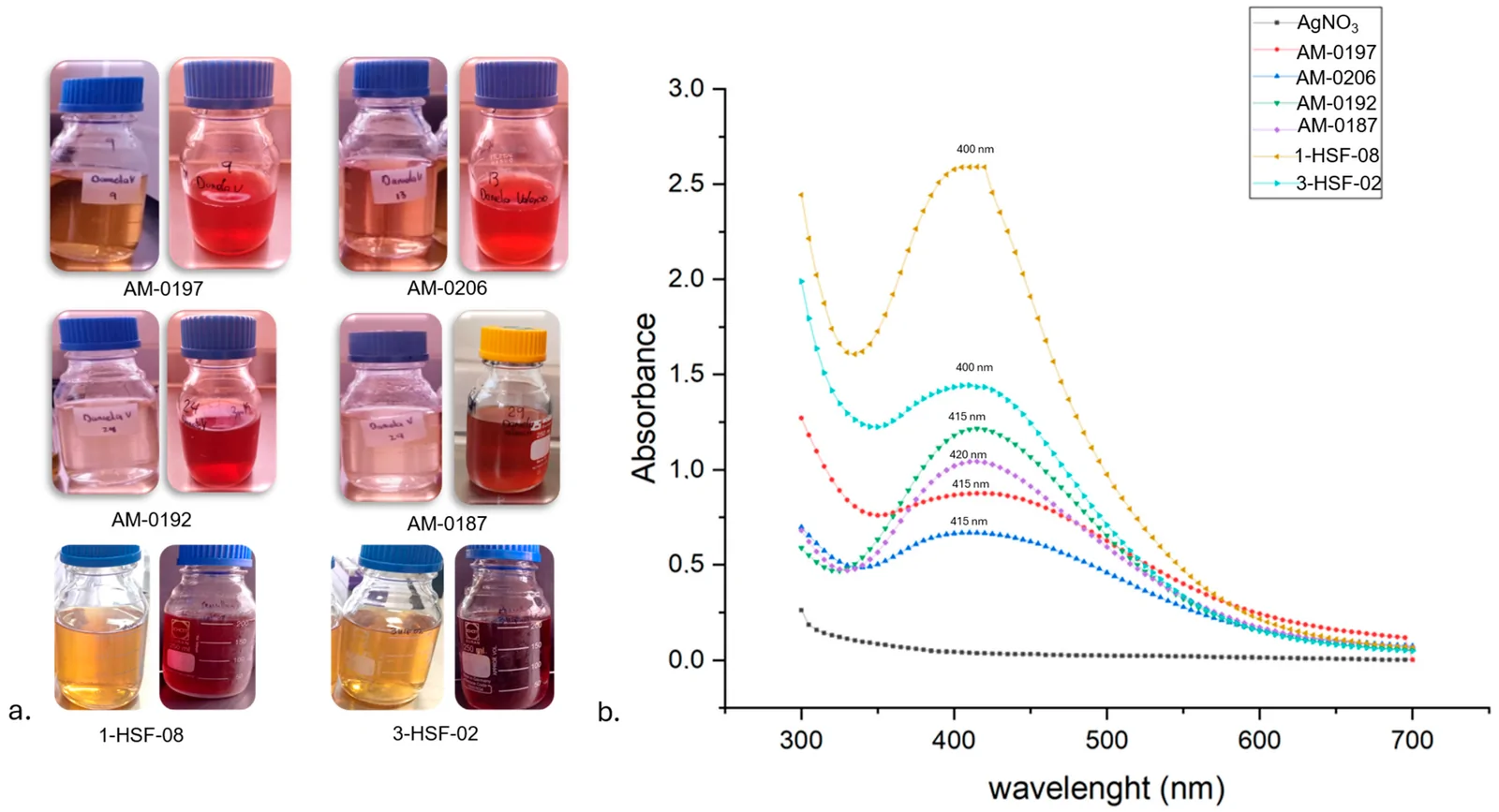
Mycosynthesis of silver nanoparticles. (**a**) Biosynthesis reactions and (**b**) UV–Vis spectra of AgNPs obtained from the six promising fungal isolates.

Silver nanoparticles were successfully biosynthesized using the six selected fungal isolates. In all cases, a color change in the reaction mixture from pale yellow to brown was observed, indicating the formation of AgNPs (Figure 2). Spectrophotometric analysis enabled the detection of the characteristic optical response of AgNPs, which is attributed to surface plasmon resonance (SPR). The appearance of this signal indicates the successful reduction of silver ions Ag^+^ and their subsequent stabilization in the reaction medium, thereby confirming AgNP synthesis.

The UV–Vis analysis showed that the maximum absorption peak of AgNPs synthesized by *Aspergillus* sp. 1-HSF-08 and *Aspergillus* sp. 3-HSF-02 appeared at 400 nm. For the nanoparticles synthesized by *F. oxysporum* (AM-0192), *F. equiseti* (AM-0197), and *F. cerealis* (AM-0206), the maximum absorption peak was observed at 415 nm, whereas the AgNPs synthesized by *Alternaria scrophulariae* (AM-0187) exhibited a maximum absorption peak at 420 nm (Figure 2).

### 3.4. Characterization of the Synthesized AgNPs

#### 3.4.1. Transmission Electron Microscopy (TEM) Analysis

**Figure 3:**
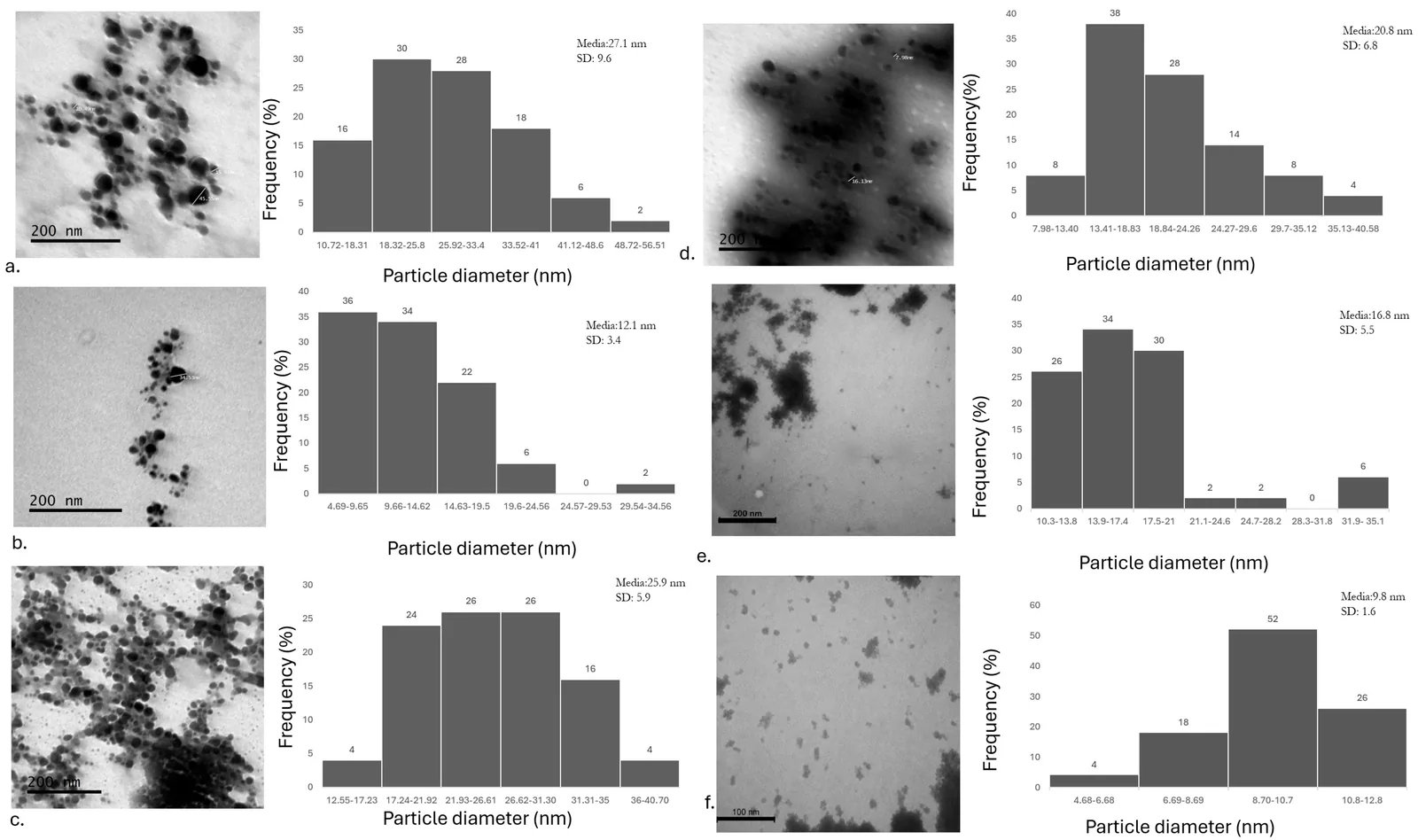
TEM micrographs and particle size distribution of the AgNPs synthesized by the promising fungal isolates. (**a**) AM-0197; (**b**) AM-0206; (**c**) AM-0192; (**d**) AM-0187; (**e**) 1-HSF-08; (**f**) 3-HSF-02. SD: standard deviation.

The detailed characterization of the size and morphology of the synthesized AgNPs was carried out by TEM. TEM micrographs revealed predominantly semi-spherical nanoparticles. The synthesized AgNPs exhibited average particle sizes between 27.1 and 9.8 nm, with the highest frequency distributions occurring within the size ranges of 18.3–25.8 nm (AM-0197), 4.69–9.65 nm (AM-0206), 21.93–31.30 nm (AM-0192), 13.41–18.83 nm (AM-0187), 13.9–17.4 nm (1-HSF-08), and 8.7–10.7 nm (3-HSF-02) (Figure 3).

#### 3.4.2. X-Ray Diffraction (XRD) Analysis

**Figure 4:**
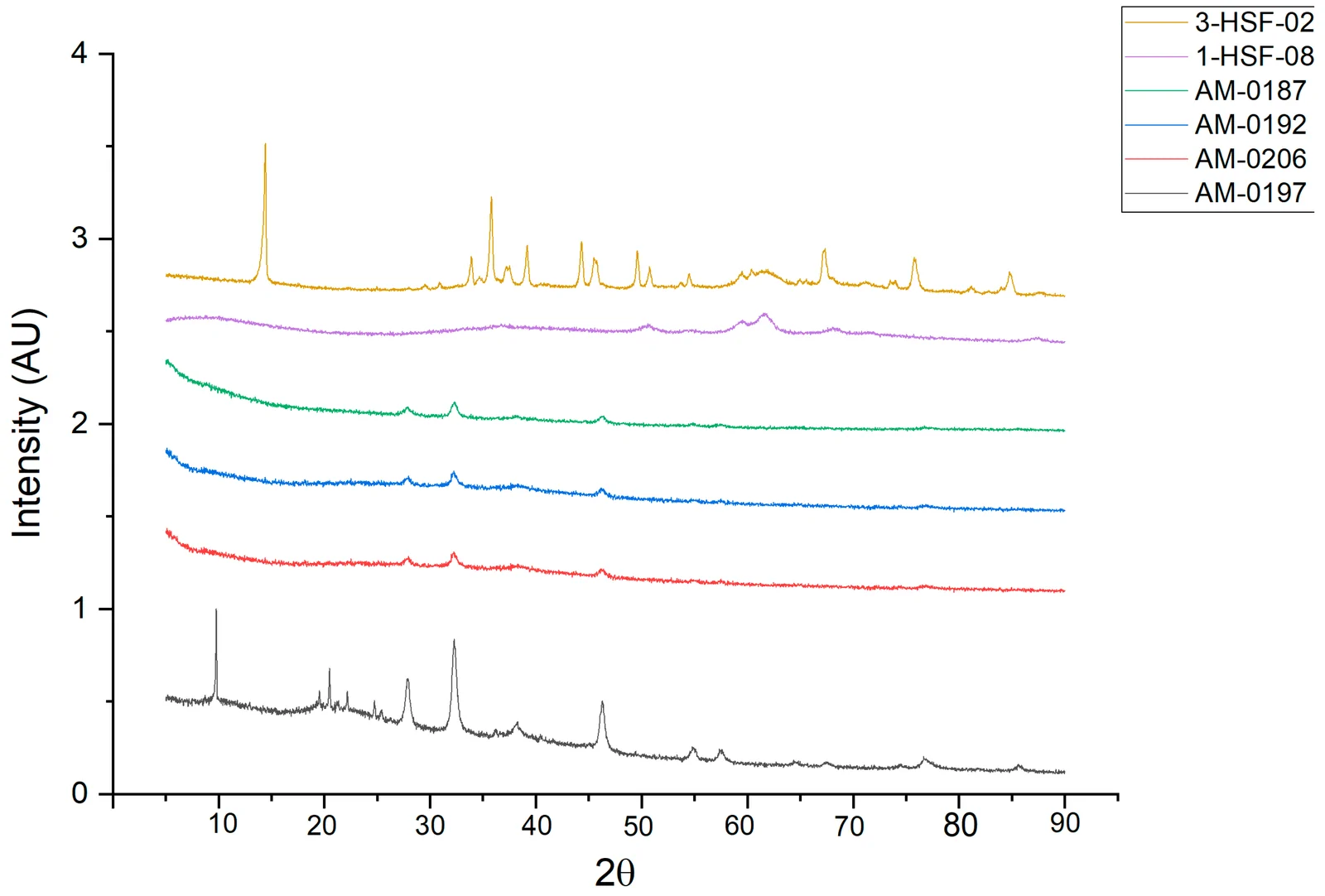
X-ray diffraction (XRD) patterns of AgNPs synthesized by the promising fungal isolates.

X-ray diffraction analysis confirmed the crystalline composition of the AgNPs synthesized by the selected fungal isolates, as evidenced by the intensity of the diffraction peaks recorded in each case [35] (Figure 4). In general, distinct diffraction patterns were observed, which are associated with the formation of spherical AgNPs exhibiting a face-centered cubic (FCC) crystal structure. All AgNPs synthesized by the three *Fusarium* isolates (AM-0197, AM-0192 and AM-0206) exhibited diffraction peaks at 2θ values of 27.7° (111), 32.5° (200), and 46.2° (200).

The AgNPs synthesized by *Alternaria scrophulariae* (AM-0187) exhibited diffraction peaks at 27.6° (111), 32.3° (200), 46.2° (200), and 57.2° (222). For the AgNPs synthesized by *Aspergillus* sp. isolates (1-HSF-08 and 3-HSF-02), diffraction peaks were detected at 9.3° (001), 19.34° (001), 20.30° (200), 22.21° (001), 27.7° (111), 32.5° (200), 36.5° (111), and 46.2° (200) (Figure 3). The mean particle diameter calculated using Scherrer’s equation was 15.7 nm for the AgNP AM-0197, 13.0 nm for AgNP AM-0206, 11.7 nm for AgNP AM-0192, 10.7 nm for AgNP AM-0187, 9.9 nm for AgNP 1-HSF-08, and 29.9 nm for AgNP 3-HSF-02.

#### 3.4.3. Fourier Transform Infrared Spectroscopy (FTIR) Analysis

**Figure 5:**
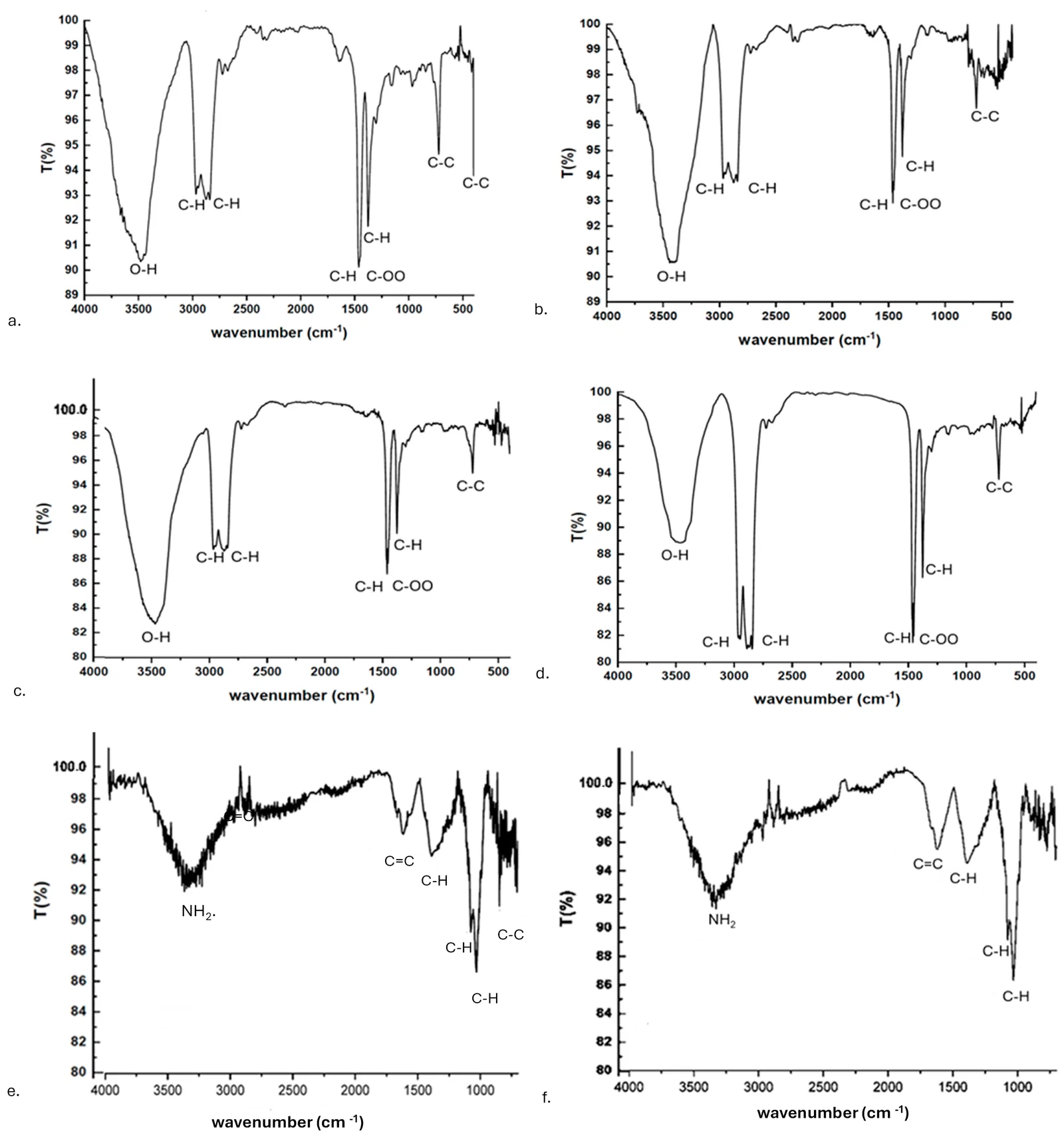
FTIR spectra of AgNPs synthesized by the promising fungal isolates. (**a**) AM-0197; (**b**) AM-0206; (**c**) AM-0192; (**d**) AM-0187; (**e**) 1-HSF-08; (**f**) 3-HSF-02.

FTIR analysis revealed that all synthesized AgNPs exhibited characteristic absorption bands corresponding to O-H (3450 cm^−1^), C-H (2900 cm^−1^), COO^−^ (1450 cm^−1^), and C-C (800 cm^−1^) functional groups, indicating the presence of organic compounds on the surface of all AgNPs (Figure 5).

#### 3.4.4. Zeta Potential and Polydispersity Index (PDI) Analysis

Negative Zeta potential values were observed for all six AgNPs evaluated. The Zeta potential values ranged from −0.4 mV for the AgNPs synthesized by *Aspergillus* sp. (1-HSF-08) to −27.7 mV for those synthesized by *F. cerealis* (AM-0206) (Table 1).

Regarding the polydispersity index (PDI), all AgNP samples exhibited values lower than 0.7. The AgNPs synthesized by *F. equiseti* (AM-0197) showed the highest degree of polydispersity, with a PDI value of 0.409, whereas the least polydisperse nanoparticles were those synthesized by *Aspergillus* sp. 3-HSF-02, which exhibited a PDI value of 0.214 (Table 1).

### 3.5. Evaluation of the Antimicrobial Activity of Mycosynthesized AgNPs Against E. coli ATCC 25922 and S. aureus ATCC 43300

**Figure 6:**
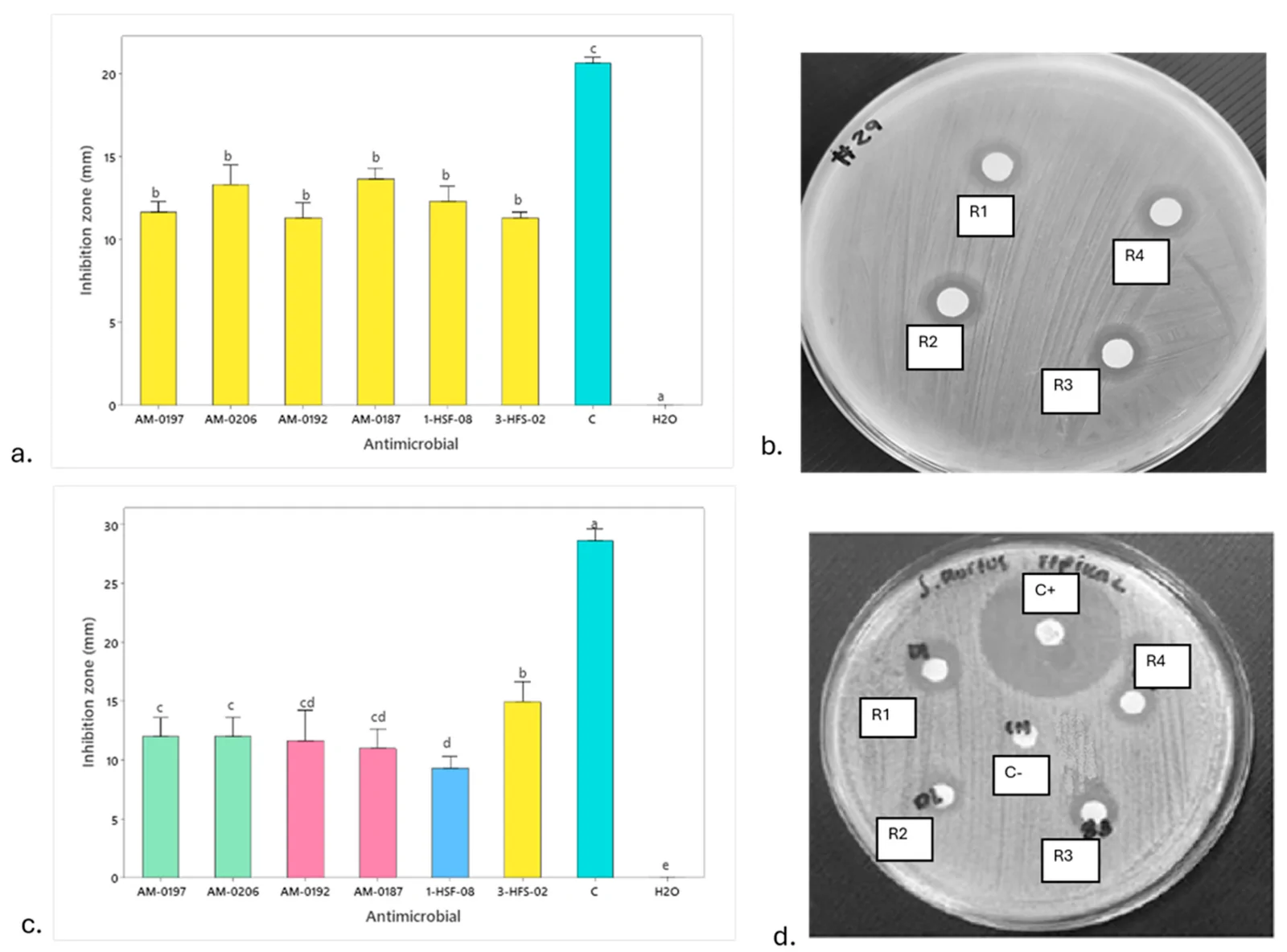
Antibacterial activity of AgNPs against *E. coli* ATCC 25922 and *S. aureus* ATCC 43300 determined by the disk diffusion assay. (**a**) Inhibition zones produced by AgNPs against *E. coli* ATCC 25922, (**b**) antibacterial activity of AgNP AM-0187 against *E. coli* ATCC 25922, (**c**) inhibition zones produced by AgNPs against *S. aureus* ATCC 43300, and (**d**) antibacterial activity of AgNP 3-HSF-02 against *S. aureus* ATCC 43300, including the corresponding controls. R1–R4 represent the replicates of the analysis. In the figures (**a**,**c**) means that do not share a common letter are significantly different according to the Tukey test at 5%.

For *E. coli* ATCC 25922, the AgNPs obtained from AM-0187 (*Alternaria scrophulariae)* exhibited an average inhibition zone of 13.6 mm, followed by AM-0206 (*F. cerealis*), which showed an average inhibition zone of 13.3 mm. In contrast, the nanoparticles AM-0197 (*F. equiseti*), AM-0192 (*F. oxysporum*), and the AgNPs 1-HSF-08 and 3-HSF-02 obtained by the genus *Aspergillus* sp. 2 exhibited smaller inhibition zones. However, no statistically significant differences were observed in the antibacterial activity of the AgNPs evaluated (*p* ≤ 0.05) (Figure 6). In this case, and as expected, the positive control of the experiment, which corresponded to the antibiotic chloramphenicol, generated the largest inhibition zones (Supplementary Table S1).

In contrast, statistically significant differences were observed in the antibacterial activity of the six AgNPs against *S. aureus* ATCC 43300 (*p* ≤ 0.05). In this case, AgNP 3-HSF-02 exhibited the highest antibacterial activity, whereas AgNP 1-HSF-08, showed the lowest inhibitory activity (Figure 6). As expected, the positive control of the experiment, which corresponded to the antibiotic trimethoprim-sulfamethoxazole, generated the largest inhibition zones (Supplementary Table S1).

### 3.6. Determination of the Minimum Inhibitory Concentration (MIC) of AgNPs Against E. coli ATCC 25922 and S. aureus ATCC 43300

Following confirmation of the antibacterial activity of the synthesized AgNPs, the minimum inhibitory concentration (MIC) was determined using the broth microdilution assay. For *E. coli* ATCC 25922, all evaluated AgNPs showed optical density (OD) values higher than those of the growth control in wells treated with 18.7 ppm AgNPs. However, at a concentration of 37.5 ppm, no turbidity was observed, and the recorded OD values were comparable to those of the uninoculated culture medium control. Therefore, this concentration was defined as the MIC for all evaluated AgNPs. Chloramphenicol, used as the reference antibiotic, also exhibited an MIC of 37.5 ppm, identical to that observed for the synthesized AgNPs.

For *S. aureus* ATCC 43300, variability in MIC values was observed depending on the AgNP evaluated. Following the same criteria, the MIC was determined to be 18.7 ppm for AgNPs AM-0197, AM-0206, AM-0192, and 1-HSF-08. The MIC of AgNP AM-0187 was 37.5 ppm, whereas AgNP 3-HSF-02 exhibited an MIC of 75 ppm, making it the least effective nanoparticle against this bacterium. Nevertheless, trimethoprim-sulfamethoxazole exhibited an MIC of 150 ppm, which was higher than those recorded for all evaluated AgNPs.

## 4. Discussion

The mycosynthesis of AgNPs has emerged as a promising alternative for the control of foodborne pathogens, as these nanoparticles exhibit high antimicrobial efficacy owing to their stability and tend to exhibit improved dispersion and controlled particle size, characteristics that are directly associated with their inhibitory activity against pathogenic microorganisms [36,37].

In the present study, the enzymatic activity of nitrate reductase was evaluated in fifty fungal isolates of environmental origin, finding that only some tested positive. This finding may be explained by the fact that nitrate reductase activity is species-specific [38]. Then, the biosynthetic potential of six environmental fungal isolates belonging to the genera *Fusarium*, *Alternaria*, and *Aspergillus* were investigated. Their ability to synthesize AgNPs is largely attributed to nitrate-reducing enzymatic activity mediated by NR, which catalyzes the reduction of Ag^+^ to elemental silver Ag^0^. The qualitative nitrate reduction assay indicated the presence of nitrites in the culture medium following incubation of the fungal isolates, providing indirect evidence of the enzymatic activity of interest [39].

The presence of NR activity was further confirmed by quantitative assays, highlighting the remarkable enzymatic capacity of the genus *Fusarium*, particularly *F. oxysporum*, which exhibited the highest nitrate reductase activity. However, despite displaying the lowest NR activity, *Aspergillus* sp. 1-HSF-08 was also able to synthesize AgNPs, suggesting that although nitrate reductase is the most extensively studied enzyme involved in mycosynthesis, it is not the sole determinant of nanoparticle formation. Anjum et al. [40] further suggested that fungi possess complex metabolic pathways capable of generating a wide diversity of secondary metabolites that play fundamental roles in nanoparticle reduction and stabilization processes. Previous studies have reported that other fungal secondary metabolites, such as anthraquinones and naphthoquinones, may participate in the reduction process due to their redox properties and may therefore be responsible for reducing the metallic precursor and promoting the subsequent formation of AgNPs [41].

Zeng et al. [42] investigated the efficient and controlled synthesis of AgNPs using a reductase isolated from *Fusarium solani* DO7, a fungus commonly found in soils and plants. To accomplish this process, two compounds were employed: β-NADPH, a cofactor required for numerous enzymatic reduction reactions, and polyvinylpyrrolidone (PVP), which acted as a stabilizing agent to control nanoparticle size and dispersion. As a result, monodisperse AgNPs with an average diameter of 9.5 nm were successfully synthesized, and the enzyme responsible for catalyzing this reaction was identified as 1,4-α-glucosidase.

In the present study, the formation of AgNPs was confirmed under all experimental conditions evaluated. This was evidenced by a color change in the reaction mixtures from yellow to different shades of brown, a phenomenon that depends on the size and concentration of AgNPs in solution. This color change is caused by excitation of the nanoparticle surface, which results in differential light absorption and scattering compared with the original filtrate [43].

It has been reported that the culture medium, fungal age, and synthesis conditions affect the morphology and size of AgNPs [44]. In this case, the potato dextrose broth culture medium used for biomass production favored the synthesis of the NR enzyme due to its high glucose content and the production of AgNPs of uniform size [45]. Using a seven-day growth biomass for the production of the mycelium-free cell extract is ideal, as this time coincides with the stationary growth phase, which favored the secretion of the NR enzyme [46]. Furthermore, the selected conditions, such as temperature, pH, and reaction time, were appropriate for promoting biosynthesis in all cases and have been reported in previous studies [27].

Some of these fungal genera have previously been reported as AgNP producers [33,47,48]. *F. oxysporum* is one of the most extensively studied fungal species in nanobiotechnology because of its ability to produce extracellular reductase-like metabolites that promote the biogenic synthesis of AgNPs [41]. In contrast, fewer studies have demonstrated this potential in the genus *Aspergillus*, and to date, no previous reports have described the ability of *Alternaria scrophulariae* to synthesize AgNPs, making this an important finding of the present study.

The spectrophotometric results obtained for the six synthesized AgNPs revealed maximum absorption peaks ranging from 400 to 420 nm, which are consistent with the findings of Liu et al. [49], who also reported absorption peaks between 420 and 430 nm for AgNPs synthesized using microbial extracts. The absorbance values observed in each mycosynthesis reaction were related to the concentration of AgNPs in solution. In this sense, it could be inferred that the fungus that would have produced the greatest amount of AgNPs would be *Aspergillus* sp. (1-HSF-08), while *F. cerealis* (AM-0206) would be the one that showed the lowest yield. However, to confirm this statement, it is necessary to quantify the reduced silver content in the solution using techniques such as ICP-Ms/OES/AAS, which was not possible to do in this investigation. This difference may presumably be attributed to higher metabolic activity and the increased production in reducing agents. Similarly, Nanda et al. reported that AgNPs synthesized by *Aspergillus tamariI* exhibited a UV–Vis absorption peak at 425 nm [50].

TEM characterization of the synthesized AgNPs revealed that all nanoparticles exhibited a semi-spherical morphology, with particle sizes ranging from 12.1 to 27.1 nm. This morphology was expected because the spectrophotometric analysis showed a single surface plasmon resonance (SPR) band in all cases, which is associated with the presence of spherical nanoparticles, whereas anisotropic AgNPs generally exhibit more than one plasmonic band [28]. These results are consistent with those reported by Husseiny et al., who synthesized *Fusarium oxysporum*-derived AgNPs with particle sizes ranging from 15 to 40 nm [51]. Likewise, Hoseini et al., working with *Alternaria* sp., obtained AgNPs with an average diameter of 14.1 nm, which was smaller than that observed in the present study for *A. scrophulariae*-derived AgNPs, which exhibited an average size of 20.8 nm [52]. However, the particle sizes obtained in the present study are comparable to those reported by Akpinar et al., who synthesized spherical AgNPs ranging from 10 to 25 nm using *Alternaria* sp [53].

Regarding the XRD analysis of the synthesized AgNPs, which allows for verification of their crystalline nature [54], all nanoparticles exhibited a face-centered cubic (FCC) crystal structure regardless of the fungal species from which they were synthesized. This crystallographic symmetry has been widely reported in studies employing fungi for the biosynthesis of AgNPs. The AgNPs synthesized by the genus *Fusarium* exhibited diffraction peaks at 2θ values of 27.7° (111), 32.5° (200), and 46.2° (200), which have previously been reported for AgNPs synthesized using the same fungal genus and correspond to the FCC structure of metallic silver [51,55,56].

The AgNPs synthesized by *Alternaria* sp. exhibited diffraction peaks at 27.6° (111), 32.3° (200), 46.2° (200), and 57.2° (222). Some of these peaks were also observed in the AgNPs synthesized by *Fusarium* spp. and correspond to the FCC structure of metallic silver. To date, these diffraction signals have not been previously reported in studies involving this fungal genus for AgNP biosynthesis [52,56].

For the AgNPs synthesized by *Aspergillus* sp. (1-HSF-08 and 3-HSF-05), diffraction peaks were observed at 32.1° (111) and 44.7° (200), which also correspond to the FCC structure and have previously been reported for AgNPs synthesized using *Aspergillus wentii* [57]. Additional peaks observed at angles below 22° are noteworthy and have been assigned to crystallized organic residues resulting from the green synthesis process and present on the surface of the AgNPs. This phase is composed of macromolecules present in the cell-free extract, which may be responsible for the reduction of silver ions during AgNP formation [52].

Biologically synthesized AgNPs are generally coated with proteinaceous molecules that constitute the so-called protein corona, which significantly influences the biological response of AgNPs because the functional groups and charge of the coating proteins regulate their cytotoxic properties [41]. To verify the organic nature of the nanoparticle surface, FTIR analysis was performed. In all cases, absorption bands associated with organic compounds were detected on the surface of the AgNPs, like those previously reported for AgNPs mycosynthesized using *Fusarium oxysporum* [57,58,59].

All synthesized AgNPs exhibited characteristic bands corresponding to C–H and C=C bonds, which are associated with the presence of aromatic hydrocarbons coating the nanoparticle surface. Gajbhiye et al. [60] and Narware et al. reported absorption peaks associated with –COO– and C–H groups in AgNPs synthesized using *Alternaria alternata* and *Alternaria solani*, respectively, similar to those observed in the present study [56].

For the AgNPs synthesized by *Aspergillus* sp. (1-HSF-08 and 3-HSF-02), a band associated with the amino (NH_2_) functional group, commonly found in amino acids, was detected. This finding suggests that proteins present in the mycelium-free cell extract may have bound to the AgNP surface through free amino groups and cysteine residues via electrostatic interactions, thereby acting as capping agents that could stabilize the nanoparticles and decrease their tendency to aggregate in aqueous media [58,61]. Additionally, an absorption band at 3450 cm^−1^ was detected in the AgNPs synthesized by *Fusarium* spp. and *Alternaria scrophulariae*, which was associated with O–H groups likely derived from carboxylic acids. Similar findings were reported by Honary et al. in AgNPs synthesized using *Penicillium* sp. [61].

The PDI is a parameter that provides information about nanoparticle size distribution and colloidal stability, with ranges from 0 to 1 and samples with broad particle size distributions generally exhibiting PDI values greater than 0.7 [59,62]. In the present study, all AgNPs samples exhibited PDI values lower than 0.7. AgNPs synthesized by *F. equiseti* showed the highest polydispersity (PDI = 0.409), whereas those synthesized by *Aspergillus* sp. 3-HSF-02 exhibited the lowest polydispersity (PDI = 0.214). Similarly, Hamedi et al., working with *F. oxysporum*, reported PDI values ranging from 0.14 to 0.27 for biosynthesized AgNPs [46].

Furthermore, all AgNPs exhibited negative Zeta potential with values up to −18.6 mV. These results are desirable because they suggest repulsive interactions between particles, thereby increasing colloidal stability [63,64,65]. Hosseni et al. [52], working with AgNPs synthesized by *Alternaria* sp., reported a Zeta potential of −20.8 mV, which was attributed to a stable colloidal system due to sufficient electrostatic repulsion preventing nanoparticle aggregation. This value is close to that obtained for the AgNPs synthesized by *Alternaria scrophulariae* in the present study. Moreover, negative Zeta potential values indicate that the AgNPs are coated with negatively charged biomolecules, which contribute to the electrostatic stabilization of the nanoparticles in suspension [28]. However, the AgNPs obtained from *Aspergillus* sp. (1-HSF-02 and 3HSF-08) showed very low Zeta potential values with values between −0.4 and −0.5, the low magnitude of the negative Zeta potential suggests limited electrostatic stabilization of the AgNPs and a potentially greater tendency toward aggregation. However, the relatively low PDI observed may indicate the contribution of non-electrostatic stabilization mechanisms, potentially associated with fungal biomolecules adsorbed onto the nanoparticle surface [66].

The antibacterial activity of the synthesized AgNPs against foodborne bacterial pathogens, evaluated by the disk diffusion assay, demonstrated that all mycosynthesized AgNPs were effective against the tested microorganisms. In the case of *E. coli*, although no statistically significant differences were found between the AgNPs evaluated, the activity of the AgNPs (AM-0187) synthesized by *A. scrophulariae* exhibited the highest antibacterial activity. Likewise, Akpinar et al. reported that AgNPs synthesized using *Alternaria* sp. exhibited potent inhibitory activity against *E. coli*, which was attributed to the smaller size of the nanoparticles, facilitating their penetration through the bacterial cell wall and promoting the generation of reactive oxygen species (ROS) and intracellular damage [53]. Balakumaran et al. evaluated the antibacterial activity of AgNPs synthesized by *Aspergillus terreus* against several pathogens, including *E. coli*, and reported inhibition zones of 3.2 mm, which are considerably smaller than those observed in the present study [63].

The AgNPs synthesized by *Fusarium* spp. also exhibited remarkable antimicrobial activity against this bacterium, consistent with previous reports. Ibrahim et al. reported inhibition zones ranging from 5 to 7 mm for AgNPs biosynthesized by *Fusarium* sp., which are smaller than those obtained in the present study [67]. These differences may be attributed to variations in nanoparticle concentration and morphology. Similarly, Soliman et al. synthesized AgNPs using *Fusarium* sp. and reported significant antibacterial activity against *E. coli*, primarily attributed to the small size and regular morphology of the nanoparticles, which favor greater contact with the bacterial membrane [68]. Morones et al. suggested that the effectiveness of AgNPs against *E. coli* is mainly associated with the release of Ag^+^ ions, which destabilize the bacterial cell wall and alter enzymatic functions [69]. Although smaller AgNPs generally exhibit greater antimicrobial activity than larger nanoparticles due to their higher specific surface area and more efficient interaction with microbial cells, this correlation was not evident in the present study. For *E. coli*, the AgNPs that generated the smallest inhibition zones were those synthesized by *F. oxysporum*; this finding is noteworthy because the antibacterial activity of AgNPs synthesized by this fungal species has been reported to be remarkable in previous studies [27,41,44,70].

For *S. aureus*, the most effective AgNPs were those synthesized by *Aspergillus* sp. (3-HSF-02), which are the smallest that were successfully synthesized in this study. Previous studies have reported that AgNPs synthesized using this fungal genus exhibit considerable antibacterial activity against this pathogen. Gade et al. investigated the antibacterial activity of AgNPs synthesized by *A. niger* against *S. aureus* and reported inhibition zones of 23 mm [71]. Likewise, Balakumaran et al. evaluated AgNPs synthesized by *A. terreus* and observed inhibition zones of 14.27 mm against *S. aureus* [63]. In contrast, Baker et al., evaluating AgNPs synthesized by *Alternaria* sp. against methicillin-resistant *S. aureus* (MRSA), reported an inhibition zone of only 3 mm, which is substantially lower than that observed in the present study [72].

The minimum inhibitory concentration (MIC) of all evaluated AgNPs against *E. coli* was 32.5 ppm, which was identical to that obtained for chloramphenicol, the positive control used in this study. However, this value was higher than those reported by Balakumaran et al. [63] and Baker et al. [72], who evaluated AgNPs synthesized by *Aspergillus terreus* and *Alternaria* sp. and reported MIC values of 3.125 ppm and 16.25 ppm, respectively. These differences may be explained by the fact that characteristics such as nanoparticle size, shape, and surface functionalization can significantly influence interactions with the bacterial surface, and consequently, antibacterial activity [70]. Furthermore, the antimicrobial activity of AgNPs has been attributed primarily to the nanoparticles themselves, whereas larger particles exert their activity predominantly through the release of silver ions [73]. Indeed, the controlled release of silver ions contributes to the sustained antimicrobial efficacy of AgNPs [74].

For *S. aureus*, the MIC values ranged from 18.7 to 75 ppm, which are higher than those reported by Biswas et al. [75], Balakumaran et al., and Baker et al., who evaluated AgNPs synthesized by *A. wentii*, *A. terreus*, and *Alternaria* sp., respectively, and reported MIC values ranging from 3.125 to 16.25 ppm using the broth microdilution assay [63,72]. Nevertheless, these MIC values were considerably lower than that obtained with the positive control, trimethoprim-sulfamethoxazole, which exhibited an MIC of 150 ppm.

In the evaluation of the antibacterial activity of solid-state AgNPs using agar plates, the antibiotic used as a positive control generated the largest zones of inhibition compared to the evaluated AgNPs. However, when this antibacterial activity was evaluated in liquid medium during the determination of the minimum inhibitory concentration, comparable activity was observed between the evaluated AgNPs and the antibiotics used as positive controls. These discrepancies could be explained by variations in AgNP concentration or differences in the diffusion rates of the nanoparticles [76,77].

In the present study, the evaluated AgNPs were more effective against *E. coli* than against *S. aureus*, which agrees with the findings of Baker et al., who reported that AgNPs are generally more effective against Gram-negative bacteria than against Gram-positive bacteria because of differences in cell wall structure [72]. Several mechanisms of action have been proposed for AgNPs, including damage to the bacterial cell wall and membrane, disruption of DNA replication and protein synthesis through interference with ribosomal function, and enzyme inactivation resulting from their affinity for thiol groups [20]. Additionally, these nanoparticles are capable of inducing the formation of reactive oxygen species (ROS), such as hydrogen peroxide (H_2_O_2_) and hydroxyl radicals (OH•), leading to oxidative stress and the disruption of multiple cellular processes, thereby enhancing their antimicrobial activity [20,27].

Furthermore, AgNPs interfere with cellular respiration and ATP production. These nanoparticles can inactivate phosphomannose isomerase, the enzyme responsible for catalyzing the conversion of mannose-6-phosphate to fructose-6-phosphate, an intermediate of glycolysis and a common pathway for sugar catabolism in bacteria, however, the primary trigger of bacterial toxicity appears to be the interference of AgNPs with ubiquinone (coenzyme Q), which participates in the respiratory process [78,79].

Finally, although cytotoxicity tests were not performed on this occasion, it has been mentioned that at low concentrations, AgNPs are not toxic to eukaryotic cells, as reported in several studies [80,81,82], which expands the possibility of using them for agricultural applications if further cytotoxicity tests demonstrate this.

## 5. Conclusions

The present study demonstrated that the selection of ambiental fungi with AgNP biosynthetic potential based on enzymatic activity constitutes an effective strategy that facilitates the identification of promising isolates; however, the enzyme cannot be the only selection criterion because in specific cases, there may not be a direct correlation between high NR activity and higher AgNP production as in the case of *Aspergillus* sp. (1-HSF-08). All synthesized AgNPs exhibited a remarkable capacity to inhibit the growth of *Escherichia coli* and *Staphylococcus aureus*, producing inhibition zones that varied according to the type of AgNPs evaluated. Although in solid culture medium the antibiotics showed the largest zones of inhibition, when determining the MIC, the values obtained for the synthesized AgNPs were equal to or lower than those observed for the antibiotics used as positive controls, reinforcing the potential of AgNPs biosynthesized by filamentous fungi as effective antimicrobial agents against foodborne pathogens. Because the possibility of bacteria developing resistance to these compounds is low given their multifaceted mechanism of action and because low concentrations of AgNPs have been reported as non-toxic to eukaryotic cells, these nanoparticles represent a promising alternative for the development of biocompatible and sustainable strategies for the treatment of bacterial infections. Nevertheless, several limitations remain. Additional studies are required to elucidate the role of fungal metabolites in controlling nanoparticle size and morphology, to improve biorecovery efficiency, and to assess the potential toxicity of these nanomaterials. These aspects should be addressed in future investigations to facilitate the development of safe and effective AgNP-based antimicrobial applications.

## Figures and Tables

**Table 1:** Zeta potential and polydispersity index (PDI) of AgNPs synthesized by promising fungal isolates.

Fungi/AgNPs	Z Potential (mV)	Polydispersity Index (PDI)
AM-0197	−18.6	0.409
AM-0206	−27.7	0.283
AM-0192	−26.0	0.257
AM-0187	−22.2	0.261
1-HSF-08	−0.4	0.285
3-HSF-02	−0.5	0.214

## Data Availability

The information on the microorganisms used in this research for the mycosynthesis of silver nanoparticles can be found in the registration cards of the collection of fungi and microorganisms of the University of Boyacá (UBCHM) registered with the National Single Registry of Biological Collections (RNC) under number 274.

## Supplementary Materials

The following supporting information can be downloaded at: https://www.mdpi.com/article/10.3390/microorganisms14092083/s1, Table S1: Antibacterial activity of AgNPs against *E. coli* ATCC 25922 and *S. aureus* ATCC 43300 determined by the disk diffusion assay.

## Abbreviations

AMR	Antimicrobial resistance
ANOVA	Analysis of variance
DAEC	Diffusely adherent *E. Coli*
DEC	Diarrheagenic *E. Coli*
EAEC	Enteroaggregative *E. Coli*
EIEC	Enteroinvasive *E. Coli*
EHEC	Enterohemorrhagic *E. Coli*
EPEC	Enteropathogenic *E. Coli*
ETEC	Enterotoxigenic *E. Coli*
FBDs	Foodborne diseases
FCC	Face-centered cubic structure
FTIR	Fourier transform infrared spectroscopy
HUS	Hemolytic uremic syndrome
MFCF	Mycelium-free cell filtrate
MIC	Minimum inhibitory concentration
MRSA	Methicillin-resistant *S. aureus*
NR	Nitrate reductase
OD	Optical density
PDA	Potato dextrose agar medium
PDB	Potato dextrose broth
PDI	Polydispersity index
ppm	Parts per million
PVP	Polyvinylpyrrolidone
ROS	Reactive oxygen species
TEM	Transmission electron microscopy
UBCHM	Fungi and microorganisms collection of the University of Boyacá
WHO	World Health Organization
XRD	X-ray diffraction
