# Supplementary material for: Mycosynthesized Silver Nanoparticles (AgNPs) for the Control of Foodborne Pathogenic Bacteria: Escherichia coli and Staphylococcus aureus
Source: Microorganisms. 2026 Sep 17;14(9):2083. doi: 10.3390/microorganisms14092083 (PMC13609474; doi:10.3390/microorganisms14092083)
Supplement: Supplementary file 1 [file microorganisms-14-02083-s001.zip › microorganisms-4459576-supplementary.pdf]

SUPLEMENTARY INFORMATION

Table S1. Antibacterial activity of AgNPs against *E. coli* ATCC 25922 and *S. aureus* ATCC 43300 determined by the disk diffusion assay

AgNPs	Zone of inhibition (mm)	
	E. coli ATCC 25922	*S. aureus* ATCC 43300
**AM-0197**	11.6±0.6 [b]	12.0±0.5 [c]
**AM-0206**	13.3±1.2 [b]	12.0±0.5 [c]
**AM-0192**	11.3±0.8 [b]	11.6±0.8 [cd]
**AM-0187**	13.6±0.6 [b]	11.0±0.5 [cd]
**1-HSF-08**	12.3±0.8 [b]	9.3±0.3 [d]
**3-HSF-02**	11.3±0.3 [b]	15.0±0.5 [b]
**Control +**	20.6±0.3 [c]	28.6±0.3 [a]
**Control -**	0.0±0.0 [a]	0.0±0.0 [e]

The mean is shown with its respective standard error. Means that do not share a common letter are significantly different according to Tukey test at 5%
